# Infectivity of *Leishmania mexicana* Is Associated with Differential Expression of Protein Kinase C-Like Triggered during a Cell-Cell Contact

**DOI:** 10.1371/journal.pone.0007581

**Published:** 2009-10-23

**Authors:** Nidia Alvarez-Rueda, Marlène Biron, Patrice Le Pape

**Affiliations:** 1 Département de Parasitologie et de Mycologie Médicale, Université de Nantes, Nantes Atlantique Universités, EA 1155 - IICiMed, Faculté de Pharmacie, Nantes, France; 2 Laboratoire de Parasitologie-Mycologie, CHU de Nantes, Nantes, France; BMSI-A*STAR, Singapore

## Abstract

Mammalian host cell invasion by *Leishmania* is a complex process in which various parasite and host cell components interact, triggering the activation of signaling cascades in both cells. Little is known regarding PKC biological functions in *Leishmania sp.* during parasite-macrophage interaction. PKC-like enzyme was first identified in homogenates and membrane fraction of *L. mexicana* stationary promastigotes by immunoblot. PKC-like enzyme activity was then detected in cell homogenates but also on intact promastigotes showing for the first time the presence of an ecto-PKC dependent on Ca^2+^/phosphatidylserine for activation. This ecto-PKC was activated with phorbol myristate acetate (PMA) and inhibited by RO-32-0432, a selective PKCαβ_I_ε bisindolylmaleimide inhibitor. Interestingly, the *Leishmania* PKC- activity was higher in the infective stationary than in non-infective logarithmic stage. Then, promastigotes at different stages of time proliferation curve were used in order to identify the role of PKC-like during macrophage invasion. After attachment to macrophages, PKC-like is over-expressed in promastigotes at the 6^th^ culture day but also at the 4^th^ day of culture corresponding to the maximal infection capacity. An antibody microarray for MAPK and PKC corroborate the *Leishmania* PKC-like over-expression during contact with macrophages. Pretreatment with RO-32-0432 inhibitor reduced the number of infected macrophages and the parasite burden. These data suggest for the first time a direct link between PKC expression level and infectivity, and provide evidence that PKC-like plays a critical role in attachment and in the internalization steps involved in the invasion process.

## Introduction

Leishmaniasis is a public health problem throughout most of the tropical and subtropical world [1], [2] and is a growing concern in war-torn countries [3]–[6]. The burden of Leishmaniasis expressed in disability-adjusted life years (DALYS) is estimated by WHO to be over 2 million [5]. Like for every parasitic diseases, there is no vaccines against Leishmaniasis, and chemotherapy is the only treatment option [7]. Disappointingly few drugs are available in clinical practice—Pentamidine, Antimonials, Amphotericin B and Miltefosine— and efficacy is limited due to the toxicity and increasing multiple drug resistance [8]–[10]. There is therefore an urgent need to identify new drug targets.

*Leishmania* parasites have a complex life cycle that renders the therapeutic approaches very difficult. Parasites move from the sandfly midgut up to the mouthparts, then into the human host where they invade macrophages and live within a phagolyzosoma. During infection of mammalian host, *Leishmania* require adaptive changes to ensure internalization and proliferation into macrophages. During internalization some components of the parasite cell surface such as gp63 and LPG are over-expressed [11]–[14]. The intermediate steps of signal transduction pathways mediating these changes are unknown. With the recent publication of the complete genome sequence of *Leishmania major* [15], a vast amount of new information will allow more comprehensive identification of parasite-specific proteins, among them protein kinases, and their biological significance.

The recent genomic analysis of *Leishmania major* shows that they possess numerous molecules suspected to bind protein kinases (PK) [16]. By comparing mammalian- and trypanosomatid-PKs, this analysis clearly indicates that PK phosphorylation is a key mechanism for the regulation of parasite processes. The understanding of the structure and function of mammalian PK is now used to elucidate the function of *Leishmania* homologues. To achieve this requires identification of structures and mechanisms that are either unique to *Leishmania* or sufficiently different to allow the identification of specific target molecules. Some of these proteins and metabolic pathways unique to *Leishmania* are under investigation [10]. In mammals, six major groups of eukaryotic protein kinases (ePK) are defined based on the sequence homology of their catalytic domain [17]. PK distribution differs between *Leishmania* and mammalian cells. First, *Leishmania* completely lacks tyrosine kinases (TK) and tyrosine kinase-like (TKL). Second, the members of AGC family such as protein kinase A, protein kinase G and protein kinase C are under-represented, but they seem to be substantially different from mammalian homologues. They could be promising drug targets in *Leishmania* [18]. Protein kinase C (PKC) represents a family of serine/threonine kinases that consist of at least 11 isoenzymes organized into three subgroups: conventional PKCs require diacylglycerol (DAG) and Ca^2+^ for activation, novel PKCs require only DAG for activation, and atypical PKCs lack responsiveness to DAG and Ca^2+^ [19]–[21]. For the moment, bioinformatic analyses of *Leishmania major* protein kinase genome have not identify a PKC orthologue (PKC-like) [22]. In the past decade, proteomic analysis of *Leishmania* promastigotes using protein kinase inhibitors and activators such as staurosporine, H7, sphingosine and TPA predicted the presence of PKC-like activity [23], [24], . However, because weakly selective PKC inhibitors were used, direct evidence of this enzyme has often been contradictory.

Activation of *L. amazonensis*-PKC modulates the interaction with host macrophages via secreted acid phosphatase [24]. In a previous work, we have demonstrated the first evidence of specific *Leishmania* PKC-like activity [26]. A recent report confirmed this PKC-like activity in *L. amazonensis* and demonstrated that it is responsible for ion homeostasis maintenance through the modulation of (Na^+^, K^+^) ATPase activity [27].

The discovery of ecto-protein kinases (ecto-PK) activity has revealed the regulatory machinery of protein phosphorylation also operates in the extracellular environment. Numerous reports demonstrate an ecto-PK activity on the surface of a number of cell types such as mammalian cells (see for review [28]) and protozoan parasites such as *Entamoeba hystolytica* and *Toxoplasma gondii* [29]. The functional significance of this extracellular phosphorylation continues to be uncovered, and reports on its implication during cell proliferation, differentiation and cell interactions have been cited [28]. We note with interest previous reports of an ecto-PK in *Leishmania*. Promastigotes possess PK at the external plasma membrane surface that is capable phosphorylating endogenous, membrane and foreign proteins [30]. Only a member of the casein kinase I (CKI) has been characterized [31]. On the other hand, extracellular protein phosphorylation in *Leishmania* increases during parasite development indicating that protein kinases can regulate host or parasite cellular processes, and their interactions [32]–[34].

In the present study, we identified for the first time an ecto-PKC in different *Leishmania* species. We demonstrated, using a monoclonal antibody recognizing PKCα,β_I_,β_II_, its differential expression during the time promastigote proliferation curve and its participation in the attachment steps of *Leishmania* internalization into macrophages as well as invasion capacity.

## Materials and Methods

### Cell and parasite cultures

*L. mexicana* (MHOM/MX/95/NAN1), *L. major* (MHOM/SEN/96/NAN2) and *L. infantum* (MHOM/FR/91/LEM2259) strains were cultured at 26°C in Schneider's insect medium (Sigma Chemical Co., St. Louis, MO, USA), supplemented with 10% fetal bovine serum (FBS, Sigma), penicillin (100 IU/ml) and streptomycin (100 µg/ml). Promastigotes were harvested at 2^nd^, 4^th^, 6^th^, and 8^th^ growth days for PKC assays. Resident peritoneal macrophages from male BALB/c mice (R. Janvier-Le Genest, France) were collected in RPMI 1640 (Sigma) culture medium supplemented with 10% FBS. Mice were handled in accordance with existing status of animal treatment as embodied in the Guiding Principles of Biochemical Research.

### Specificity of anti-PKC antibodies

To date no data are available on the amino acid and/or nucleotide sequences of *Leishmania* protein kinase C. The available information from the *L.major* genome database has not been unambiguously identified a PKC ortholog. We therefore used multiple data sources to infer the specificity of the antibodies used in this study: Western blots and BLAST searches in the *Leishmania* genome for the amino acid sequences (at http://www.genedb.org/genedb/leish/blast.jsp/) and the sequence alignments (with http://www.ch. embnet.org/software/LALIGN_form.html and http://bioinfo. genopole-toulouse.prd.fr/multalin/multalin.html). We also “BLASTed” the proteins retrieved from the *Leishmania* database against all genus kinomes, in order to check for protein similarities among species.

To choose good candidates for *Leishmania* PKC-like among the BLAST hits, we adopted a stringent significance level taken into account the molecular weight of matched proteins, the scores and E-values (the cutoff was E<200), because the chance of obtaining random matches with short amino acid sequences was relatively large.

### *Leishmania* homogenate and membrane preparations

Promastigotes of *L. mexicana* were harvested by centrifugation at 2500 *g* for 10 minutes at 4°C. Cell pellets were suspended and washed in phosphate buffer saline (PBS) pH 7.2. Subsequent procedures were performed at 4°C. Packed cells (2.5×10^9^ cells/ml) were suspended in 0.02 M Tris-HCl containing 0.25 M sucrose and 3 mM MgCl_2_. Cells were homogenized and sonicated in a Branson homogenizer 250 W. After centrifugation at 1000 *g* for 10 minutes, the supernatant was adjusted with 0.1 mM phenylmethane sulphonyl fluoride (PMSF), 0.02 mg/ml leupeptin and 0.05 mg/ml aprotinin. The resulting suspension was further centrifuged at 100000 *g* for 1 hour and the membrane fraction (pellet) was separated from the supernatant. Protein concentration was determined by the Bradford method [35] using bovine serum albumin (Sigma) as standard. Homogenate and membrane fraction were directly used for enzyme activity detection or stored at −80°C until use for immunoblot analysis.

### Protein kinase C-like activity

Protein kinase C-like activity was measured by an enzyme-linked immunosorbent assay, ELISA (Calbiochem, San Diego, CA). A synthetic PKC pseudosubstrate (RFAARKGSLRQKNV) and a monoclonal antibody (2B9) that recognizes the phosphorylated form of the peptide were used for PKC activity detection. Intact promastigotes (2 mg protein/ml), total homogenate (500 µg protein/ml) and membrane fraction (500 µg protein/ml) were suspended in a total volume of 100 µl of mixture buffer containing 25 mM Tris-HCl, pH 7.0, 3 mM MgCl_2_, 0.1 mM ATP, 2 mM CaCl_2_, 50 µg/ml phosphatidylserine, 1 mM EGTA, 0.5 mM EDTA, 0.1 mM PMSF, 1 mM benzamidine. After 30 min. incubation at 30°C, biotinylated antibody 2B9 was added to each well. PKC activity was revealed by a peroxidase-conjugated streptavidin antibody and by using the ortho-phenyldiamine as substrate. Plates were read at 492 nm. PKC activity was expressed in international units (IU/mg) using a PKC control (2000 U/mg, Promega, USA) as positive control. In some experiments, intact promastigotes were pretreated with the PKCαβ_I_ε inhibitor, RO-32-0432 (Calbiochem) [36] for one hour at concentrations between 0.0001 and 1 µM. The PKC activator phorbol myristate acetate (PMA) was used during 15 min at concentrations between 0.035 and 1 µg/ml. Promastigote mobility was monitored after treatments by light microscopy before measuring the PKC-like activity.

### Immunoblot analysis

Promastigotes were washed in PBS pH 7.2 and centrifuged at 2500 g for 10 minutes. Cell pellets were suspended in 0.02 M Tris-HCl containing 0.25 M sucrose and 3 mM MgCl_2_, homogenized and centrifuged at 14000 g for 15 minutes to eliminate cell debris. Whole homogenate protein and membrane fractions (40 µg/well) were boiled in a SDS-PAGE electrophoresis according to Gallagher method [37], using 10% (w/v) polyacrylamide gels. Gels were fixed and silver-stained (Sigma). Proteins were also transferred to 0.45 µm-nitrocellulose membranes (Millipore, Bedford, USA), blocked with PBS-5% milk overnight and incubated for 2 hr in 0.05% Tween Tris-HCl pH 7.2, containing 1 µg/ml of mouse monoclonal anti-PKCα,β_I_,β_II_ (Sigma). After washes, membranes were incubated with anti-IgG-peroxidase conjugate (1/3000) (Sigma) for 2 hours. Membrane-bound proteins were revealed by chemiluminescent detection using luminol as substrate (Boehringer Mannheim, Germany). In some experiments, promastigotes were pretreated with 250 ng/ml of PMA during 15 min at room temperature before homogenization.

### *Leishmania*-macrophage internalization assay

Adherent BALB/c peritoneal macrophages were cultured at 37°C in RPMI 1600 medium supplemented with 10% fetal bovine serum and antibiotics. After 3 washes, *L. mexicana* promastigotes (2×10^6^ cells/ml) were added to 24-wells microplates containing adherent macrophages (parasite∶macrophage ratio, 2∶1). After an 18 hours contact, macrophages were washed and stained with May-Grünwald-Giemsa. The number of infected macrophages and the parasite load were calculated observing 100 macrophages/well. Results were expressed as the percentage of inhibition of these two parameters in comparison to the controls and were analyzed using the Student's *t*-test. In some experiments, promastigotes were pretreated for 1 hour with the PKC inhibitor (RO-32-0432) at concentrations between 0.0001 and 1 µM before the contact with macrophages.

The participation of PKC activity in the attachment step of the internalization process was studied during *L. mexicana* proliferation by incubating promastigotes harvested at 2^nd^ (middle log-growth phase), 4^th^, 6^th^, and 8^th^ growth days with adherent peritoneal macrophages. Briefly, adherent BALB/c peritoneal macrophages were cultured at 37°C in RPMI 1600 medium supplemented with 10% fetal bovine serum and antibiotics. After 3 washes, promastigotes (2×10^6^ cells/ml) were added to microplates containing adherent macrophages (parasite∶macrophage ratio, 2∶1). After 3 hours contact, attached but not internalized promastigotes were recovered by gently media aspiration. After 3 washes, parasites were counted before lysis. Macrophages contamination was not observed after light microscopy monitoring. Promastigotes were homogenized in the Western blot buffer before immunoblot analysis.

### Antibody MAPK and PKC microarray assay

*L.(L). mexicana* promastigotes (4^th^ culture day) were incubated with murine BALB/cmacrophages in the same conditions described above for the investigation of PKC-like activity during the attachment step. Promastigotes without macrophages were incubated in the same conditions and used as control. The assay was performed as described in Panorama Ab Microarray MAPK and PKC pathways kit according to the manufacturer's instructions (Sigma). Briefly, harvested and washed promastigotes were lysed in the Buffer A. 500 µg/ml of protein extract from control and macrophage-incubated promastigotes were labeled with Cy5 or Cy3, respectively. Free non-incorporated Cy3 and Cy5 dyes, were separated by applying the labeled extracts on SigmaSpin post-reaction clean-up columns. An equal amount of labeled proteins (30 µg) was incubated on the Panorama Ab microarray slides for 2 hours. After one wash with 0.01 M PBS pH 7.4, the slides were air-dried before scanning with the ScanArray Gx (PerkinElmer). Images were generated with the GenePix software and data from duplicate spotted protein binding were normalized with respect to anti-actin binding. Also included in the analysis were the data from two dye-swapped replicates, in which the samples were reverse color labeled.

## Results

### Antibody specificity

To choose good candidates for *Leishmania* PKC-like among the BLAST hits, we adopted a stringent significance level taken into account the molecular weight of matched proteins, the scores and E-values (the cutoff was E<200), because the chance of obtaining random matches with short amino acid sequences was relatively large.

As summarized in Table 1, the anti-PKCα,β_I_,β_II_ antibodies used in this study were raised against polypeptides that yielded homology hits when “BLASTed” against the *Leishmania* database. One homolog was found for putative protein kinase (Q4FYY9). Five other matches corresponded to hypothetical proteins. Among these uncharacterized proteins, two were found to be homologous of mammalian AGC protein kinases (Q4QCI2 and Q4QGQ8).

**Table 1:** BLAST (basical alignment search tool) results and relative molecular mass (Mr) of putative protein kinase C in the *Leishmania* genome database.

Asccession number	Protein Name		Sequence comparison	Identity (%)	Positives (%)	E-value	Length (aa)	Mass (Mr)
Q4Q0K8	Hypothetical protein	Source	3 NPQFVHPILQSA 14					
		Consensus	NP FVH I+ +A	58	75	25	656	78
		Leishmania	497 NPPFVHEIIAAA 508					
Q4Q5A1	Hypothetical protein	Source	3 NPQFVHPILQSA 14					
		Consensus	NP+ +H +LQSA	58	83	85	654	72
		Leishmania	527 NPE-MHAVLQSA 537					
Q4FYH3	Hypothetical protein	Source	6 FVHPILQSAV 15					
		Consensus	FVH +LQ AV	70	80	110	853	93
		Leishmania	414 FVHRLLQRAV 423					
Q4FYY9	Protein kinase putative	Source	6 FVHPILQS 13					
		Consensus	F HPILQ+	75	87	130	982	107
		Leishmania	890 FYHPILQA 897					
Q4QCI2 (*)	Hypothetical protein	Source	2 GNVELRQKFEKAKLGPAGN 20					
		Consensus	G +E++Q ++KA G +G+	36	73	42	1066	114
		Leishmania	586 GQLEVQQVYDKAFSGSSGH 604					
Q4QGQ8 (*)	Hypothetical protein	Source	11 EKAKLGPAGNKV 22					
		Consensus	EK GP G K+	50	58	26	562	60
		Leishmania	463 EKTTTGPNGQKI 474					

(*) Uncharacterized proteins with homology for AGC kinases in the Kinases genome database.

In summary, we feel confident about the specificity of the commercially available anti-PKC peptide antibodies, since the mammalian and *Leishmania* homologs matched with putative protein kinases, and the molecular masses of *Leishmania* and mammalian homologs are similar.

### *Leishmania mexicana* PKC-like in stationary promastigotes

**Figure 1:**
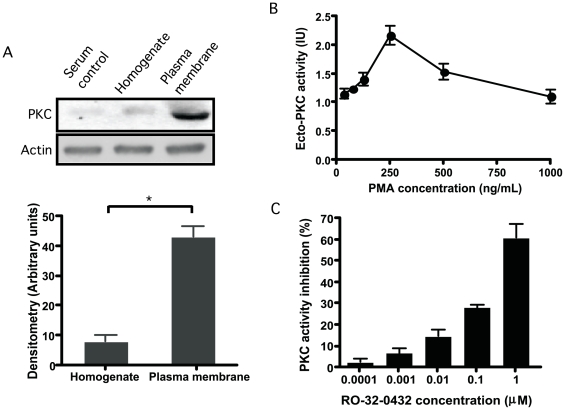
Protein kinase C-like detection on stationary promastigotes. (A): Immunoblot detection of PKC-like in subcellular fractions of stationary promastigotes of *L. mexicana*. Cell pellets (2.5×10^9^ cells/ml) were resuspended in 0.02 M Tris-HCl supplemented with 0.25 M sucrose and 3 mM MgCl_2_. Cells were homogenized and sonicated in a Branson Sonifier 250 W, then resuspended in a buffer containing protease inhibitors and centrifuged at 100000 *g* for 1 hour at 4°C. Membrane fraction (pellets) and whole homogenates were directly used for immunoblot detection of PKC using a mouse monoclonal anti-PKCα,β_I_,β_II_ antibody. (B): Ecto-PKC-like activity on intact stationary promastigotes of *Leishmania.* Promastigotes of *L. mexicana* were resuspended in 25 mM Tris-HCl, pH 7.0, 3 mM MgCl_2_, 0.1 mM ATP, 2 mM CaCl_2_, 50 µg/ml phosphatidylserine, 1 mM EGTA, 0.5 mM EDTA, 0.1 mM PMSF, 1 mM benzamidine. Phorbol myristate acetate (PMA) was added to cell suspensions in concentration ranging from 0.035 to 1 µg/ml and incubated for 15 min. at 30°C. After washes, promastigotes were incubated for 30 min at 30°C with the biotinylated antibody 2B9. PKC activity was revealed by a peroxidase-conjugated streptavidin antibody coupled to ortho-phenyldiamine substrate. Plates were read at 492 nm. Results of three different experiments were expressed in international units (IU/mg) of PKC ± SD. (C): Ecto-PKC-like inhibition. In some experiments intact stationary promastigotes were pretreated with the PKCαβ_I_ε inhibitor RO-32-0432 in concentration ranging from 0.0001 to 1 µM during 1 hour at 26°C. PKC activity was revealed by incubating with the biotinylated antibody 2B9. Results of three different experiments were represented as the percentage of PKC activity inhibition ± SD.

*L. mexicana* promastigotes from the 5^th^ proliferation day were analyzed by immunoblot. The presence of a PKC-like enzyme was detected using an specific antibody for a consensus region of conventional isotypes of PKC (α, β_I_, β_II_). Under native conditions monoclonal antibody recognizes, in homogenate and membrane fraction, a band of approximately 78 kDa corresponding to the molecular mass of the mammalian PKC detected by this antibody. Densitometric analysis showed that PKC protein level was significantly higher in the plasma membrane fraction compared to the homogenate counterpart (Figure 1A).

PKC-like activity was then evaluated by ELISA, using intact promastigotes, to investigate a putative ecto-PKC activity. Whole cell homogenates and intact parasites from stationary *L. mexicana* promastigotes were compared after incubation with the pseudosubstrate (RFAARKGSLRQKNV) in the presence of Ca^2+^ and phosphatidylserine for activation. The PKC activity was revealed using a monoclonal antibody (2B9) that recognizes the phosphorylated form of the peptide. PKC-like activity in homogenates and on intact promastigotes (ecto-PKC) was 6.3 IU/mg ±5.4 and 1.37±0.15 IU/mg, respectively. These results confirm that *L. mexicana* expresses a Ca^2+^/phosphatidylserine-dependent PKC-like protein also in the external face of plasma membrane.

Intact stationary promastigotes of *L. mexicana* were then treated with various concentrations of PMA activator (0.35 to 1 µg/ml) during 15 minutes and PKC activity was measured at the end of the experiment. At these concentrations, PMA has not effect on promastigote mobility after light microscopy observation. As shown in Figure 1B, PMA treatment caused an increase of *Leishmania* ecto-PKC activity that reached a maximum at 250 ng/ml PMA. The ecto-PKC activity was confirmed by incubating a selective bisindolylmaleimide PKC inhibitor (RO-32-0432). Pre-treatment of intact stationary promastigotes of *L. mexicana* with the PKC inhibitor at concentrations ranging from 0.0001 to 1 µM during 1 h at 26°C reduced the enzyme activity (72%±0.2 at 50 nM, p>0.002) in a dose-dependent manner (Figure 1C). At these concentrations, RO-32-0432 has not effect on promastigote mobility after light microscopy observation.

This ecto-PKC activity was also detected on intact promastigotes of other *Leishmania* species such as *L. infantum* and *L. major* (0.69±0.04 and 0.66±0.06 IU/mg respectively).

### *Leishmania* PKC-like expression during proliferation cycle

**Figure 2:**
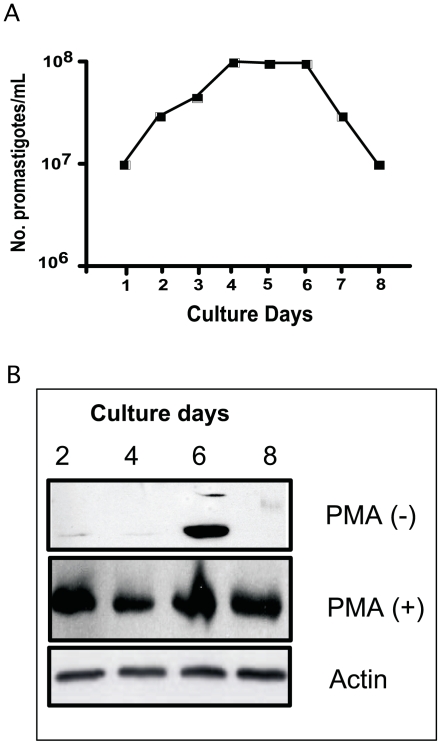
*Leishmania* PKC-like expression during proliferation. (A): Time proliferation curve of promastigotes. Parasites were cultured at 26°C in Schneider's insect medium supplemented with 10% of fetal bovine serum and antibiotics. Promastigotes were harvested at the 2^nd^, 4^th^, 6^th^, and 8^th^ growth days and counted on a microscope. (B): Immunoblot analysis of *L. mexicana* promastigotes at different stages of proliferation. 1.10^7^ promastigotes (40 µg of protein/well) were tested in a 10% SDS-PAGE gel electrophoresis. PKC was detected using 1 µg/ml of an antibody directed to a consensus sequence of mammalian PKCα,β_I_,β_II_.

In order to discriminate between the logarithmic and stationary growth phase of cultures, cell proliferation was measured by cell counting. The logarithmic-growth phase was from the 1^st^ to the 3^rd^ culture day followed by a stationary-growth phase between the 4^th^ and the 7^th^ culture days (Figure 2A).

We were then interested in describing how the PKC-like activity and level could change during the time proliferation curves of *L. mexicana* promastigotes. The ecto-PKC activity was first compared between non-infective logarithmic (2^nd^ day) and infective-stationary promastigotes (5^th^ day) of *L. mexicana*. Baseline levels of ecto-PKC activity were higher in the stationary (1.37±0.15 IU/mg) than in logarithmic stage of promastigotes (0.89±0.2 IU/mg).

Promastigote homogenates harvested at different times in the proliferation curve, were analyzed by immunoblot. The gels were loaded with the same amount of protein for each proliferation stage and immunoblot revealed by the mouse monoclonal anti-PKCα,β_I_,β_II_ (Sigma) an anti-beta actin antibody (Sigma) as control. Results from three different experiments indicated that the relative levels of PKC depend on promastigote stage of proliferation. As shown in Figure 2B, levels of PKC were extremely low at the 2^nd^ (middle logarithmic-growth phase) and 4^th^ (early stationary growth phase) days, whereas a marked increase in enzyme expression was detected only at the 6^th^ growth day (middle stationary-growth phase). Interestingly, pretreatment of promastigotes with 250 ng/ml PMA during 15 min leads to a substantial increase of PKC protein levels in all proliferative stages.

### Implication of PKC-like in the internalization process

#### Western blot analysis

**Figure 3:**
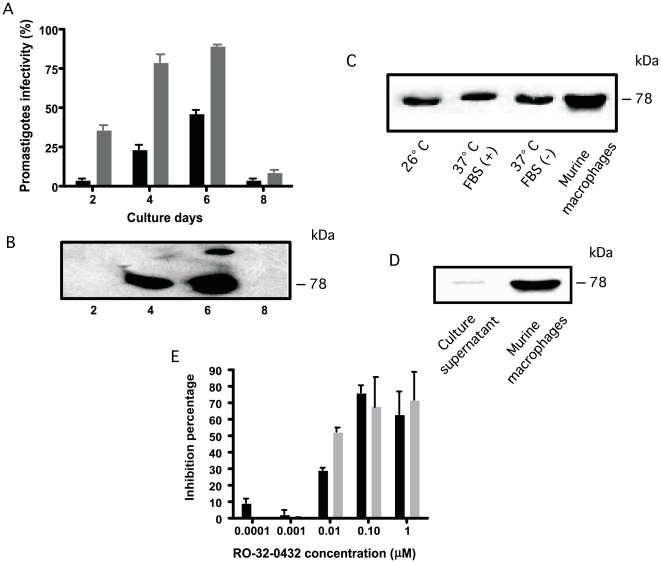
Implication of *Leishmania* PKC-like during macrophage internalization. (A): Internalization assay. Promastigotes at the 2^nd^, 4^th^, 6^th^, and 8^th^ growth days were put in contact with adherent murine macrophages in a 2∶1 parasite∶macrophage ratio and incubated at 37°C, 5% CO_2_ for 18 hours. Cells were stained with May-Grünwald-Giemsa and the number of infected macrophages (*black column*) and the parasite load (*gray column*) were calculated observing 100 macrophages/well. Results were expressed as the percentage of inhibition of these two parameters in comparison to the controls and were analyzed by using Student's *t*-test. (B): Expression of parasite PKC during internalization into macrophages. The participation of PKC activity in the attachment step of internalization process was studied during *L. mexicana* proliferation by incubating promastigotes harvested at the 2^nd^ (middle log-growth phase), 4^th^, 6^th^, and 8^th^ culture days with adherent peritoneal macrophages. After a 3 hr contact period, promastigotes were harvested, lyzed and tested for PKC in a SDS-PAGE gel electrophoresis under native conditions using the monoclonal antibody directed to a consensus sequence of mammalian PKCα,β_I_,β_II_. (C): *L. mexicana* promastigotes at 4th growth day were incubated at 26°C or 37°C in absence or the presence of 10% FBS. (D): promastigotes were incubated only with the macrophages culture supernatant or with the macrophages during 3 hr before electrophoresis. (E): Effect of PKC inhibition on *L. mexicana* promastigote internalization. Stationary promastigotes of *L. mexicana* (2x10^6^ cells/ml) were pretreated for 1 hr with the selective PKC inhibitor (RO-32-0432) at different concentrations. After washes, promastigotes were added to 24-wells microplates containing adherent macrophages (parasite∶macrophage ratio, 2∶1). After 18 hour later macrophages were stained with May-Grünwald-Giemsa. The number of infected macrophages (*black column*) and the parasite load (*gray column*) were calculated observing 100 macrophages/well. Results were expressed as the percentage of inhibition of these two parameters in comparison to the controls and were analyzed by Student's *t*-test.

Participation of PKC-like during *Leishmania* attachment step of the internalization process into macrophages was studied in an *in vitro* model of BALB/c macrophages infected with *L. mexicana* promastigotes. In this model, the capacity of promastigotes to infect macrophage increased during the time proliferation curve as represented by the percentage of parasitized macrophages and the number of intracellular parasites (Figure 3A). Precisely, the significant capacity of infection was at the 4^th^ and the 6^th^ days.

Then we compared promastigote PKC-like expression at different proliferation stages after 3 hours of contact with peritoneal BALB/c macrophages to ensure parasite attachment. Immunoblot analysis showed that after attachment to macrophages, PKC-like is over-expressed in promastigotes at the 6^th^ day but also at the 4^th^ day culture in comparison to the previous basal PKC-like expression (Fig. 3B). In order to evaluate whether culture conditions have an effect on PKC-like expression, the same experiment was developed at different temperatures (26°C or 37°C) and in the presence or absence of 10% fetal bovine serum (FBS). As shown in Figure 3C, over-expression of parasite PKC-like after incubation with macrophages depended neither on promastigotes heat shocked after passage in macrophage culture conditions nor on FBS stimulation, but rather on the cell-cell contact process. In the same conditions, macrophage-secreted molecules in the culture supernatant did not have an effect on promastigote PKC-like expression (Figure 3D).

*Leishmania* PKC-like involvement in promastigote internalization into BALB/c macrophages was further studied after treatment with RO-32-0432 inhibitor for 1 hour. Figure 3E shows that 100 nM RO-32-0432 leads to a significant reduction in the number of infected macrophages (50%, p<0,004) and parasites per infected macrophages (72%, p<0,004). These results indicated that PKC inhibition reduced capacity of *Leishmania* to internalize into murine macrophages.

#### Antibody microarray analysis

Variation in promastigote signaling protein expression after incubation with macrophages was also analyzed by the Panorama Ab microarray. This microship composed of 84 different antibodies directed against kinases and phosphatases of the MAPK and PKC pathways allowed the detection of specific promastigote protein binding in spite of the use of antibodies against mammalian proteins. 74 promastigote proteins were specifically recognized on the ship. Only 4 proteins were considered as significantly differentially expressed. A increase in the PKC-like expression in promastigotes after macrophage contact was confirmed by this method (1.64-fold increase). A stress activating protein kinase kinase 2 (SKK2, 1.6-fold increase), stress activated protein kinase 3 (SAPK3, 1.33-fold increase) and serine-theronine protein phosphatase 2Cαβ (1.23-fold increase) were also associated to promastigote attachment. Non-proteins were found to be significantly down-regulated after promastigote contact with macrophages.

## Discussion

During *Leishmania sp.* life cycle the parasite passes between the sandfly vector and the mammalian host, oscillating between replicative forms (promastigote in the sandfly and amastigote within mammalian macrophage cells) and the cell cycle arrested metacyclic form, which corresponds to the mammalian-infective form found within the mouthparts of the sandfly [38], [39]. This later corresponds to the promastigote in stationary-growth phase. Thus there appears to be an important link between the parasitic life cycle and its biological development cycle. During these morphological and metabolic changes, protein kinases are key regulatory molecules. In fact, changes in phosphoprotein abundance in *Leishmania* have been documented throughout their life cycle [32]–[34], but their significance for parasite differentiation and virulence remains unclear to date. The recent bioinformatics analyses of the *L. major* genome did not clearly identify a PKC orthologue [22]. Nonetheless, several lines of evidence suggest that a *Leishmania* PKC-like activity is present in promastigotes and its biological significance is different from the mammalian counterpart. First, protein phosphorylation increases during *Leishmania* proliferation, differentiation and interaction with host cells [32]–[34]. Second, proteomic analyses of *Leishmania* promastigotes by using protein kinase inhibitors and activators such as staurosporine, H7, sphingosine and TPA predicted the presence of a PKC-like activity [23]. Third, *Leishmania* PKC-like have been associated with biological properties such as interaction with host macrophages [24] and maintenance of ion homeostasis [27]. Thus, we investigated whether *Leishmania* PKC-like could participate during invasion of host macrophages.

Similar to mammalian cells, stationary *L. mexicana* promastigotes present a soluble and a membrane-bound form of enzyme [40]. Using a monoclonal antibody recognizing an epitope located within the amino acid sequence 296–317 at the hinge region of PKC, immunoblot analysis showed that levels of PKC are more important in plasma membrane compared to promastigote homogenates. The putative presence of an ecto-PKC was further investigated by measuring the phosphorylation by intact promastigotes of a synthetic PKC pseudosubstrate in the presence of Ca^2+^ and phosphatidylserine. We showed here the first evidence that an ecto-PKC-like activity is present in surface membrane of *L. mexicana*, *L. infantum* and *L. major* promastigotes. This enzyme activity was dose-dependently inhibited by RO-32-0432, a specific inhibitor of the bisindolylmaleimide family [41] and increased by the PMA activator. These results are in contrast to the first biochemical studies that showed inhibition of substrate phosphorylation by using PKC inhibitors such as sphingosine, currently questioned because of their poor selectivity [42].

Our results are in accordance with several studies that demonstrated the presence and activity of membrane externally PK on *Leishmania* promastigotes that is capable of phosphorylating endogenous membrane substrates and foreign proteins [25]. Similar to *Trypanosoma cruzi* and *Trypanosoma brucei* PKC, the activity of PK is Ca^2+^/phophatidylserine-dependent [43], [44]. In *L. donovani* promastigotes, a cyclic-nucleotide-independent protein kinase has been characterized in the outer surface of the parasite [23]. However, *L. mexicana* ecto-PKC detected in this work has different requirements for activity.

In a second part of our work, we investigated the possible changes in PKC activity and expression during the time proliferation curve with a particular attention to the infective stationary stage. Our results indicated an higher ecto-PKC activity in infective stationary stage. Other studies have been reported that *Leishmania* extracellular protein phosphorylation increased during parasite development. We provide also the first evidence of PKC-like level increase during proliferation with a higher PKC expression at the 6^th^ day. Expression of PKC can be induced in all stages of the time proliferation curve after a brief pretreatment with phorbol esters such as PMA. Protein phosphorylation by PKC during cell growth was previously demonstrated for other protozoan parasites such as *Trypanosoma cruzi*, *Trypanosoma brucei* and *Entamoeba histolytica* [43], [44], [45]. However, this PKC expression increase was not strictly correlated with the promastigote infective capacity. Indeed, as in non-infective logarithmic parasites, PKC expression of promastigotes harvested in the early infective stationary growth phase (4^th^ day) was very low whereas on this day, the promastigotes are fully infective. It is important to note that during the attachment to BALB/c macrophages, the first step for invasion, PKC over-expression was induced in these early stationary promastigotes (4^th^ day).

Next we examined the capacity of pre-treated parasites with RO-32-0432 to invade host cells. We first demonstrated that *L. mexicana* PKC modulates the parasite internalization and replication into BALB/c macrophages. Another research group evidenced that pre-treatment with a PKCαβγ inhibitor (RO31-8220) reduces both parasite infectivity and growth [46]. In addition, the pre-treatment of *Toxoplasma gondii* tachyzoites with 500 nM PMA enhances parasite infectivity. The TPA pre-treatment of *Leishmania* promastigotes before parasite-macrophage interaction enhances invasion process [24].

The role of ecto-PKC during differentiation from promastigote to amastigote is a boundary that has never been clearly established. An increase of protein phosphorylation during the transition promastigote to axenic amastigotes was the first observation supporting the idea that protein kinases are presented into the two stages of *Leishmania* cell cycle [34], [47], [48]. For instance only one ecto-serine/threonine protein kinase have been characterized in *Leishmania*, the ecto-casein kinase 1, LCK1 [30]. It is expressed in all the stages of the life cycle, enhanced when switches to amastigote form. These studies showed not evidence that LCK1 activity was related to PKA or PKC. Activators and inhibitors of these enzymes had no significant effect on the phosphorylation of exogenous or endogenous LCK1 substrates.

More recently other laboratories have proposed that the melting down of the flagellum during promastigote differentiation into amastigotes, is a process similar to autophagy [49]. This transition seems to be regulated by coordinated action of protein kinases and phosphatases. In the light of our results, the ecto-PKC over-expression during macrophage contact might provide some clues regarding a potential preadaptation of parasite for survival within the macrophage.

Using antibodies specific to mammalian mitogen-activated protein kinases (MAPK) and PKCs in a microarray approach, we assessed the expression changes of promastigotes proteins associated with macrophage attachment. The PKC-like over-expression was confirmed by this method. SKK2 (MKK3, MEK3, SAPKK2), a stress activating protein kinase kinase 2 was also over-expressed. Two other proteins, SAPK3 (p38γ) and serine-threonine protein phosphatase 2Cαβ, showed less expression changes. These results let to think these MAPKK and MAPK homologues in *Leishmania* also participated during the attachment process.

In *Leishmania*, MAPK-like kinases have been identified [50]–[52], and their significance in parasite flagellar morphology, virulence and survival has been studied in genetic null mutant studies. We note with interest a previous work showing the presence of a conserved MAPK kinase kinase displaying sequence homology with the mammalian SKKs [50]. This kinase designated LmxMKK, activates LmxMPK3 during the expression of flagellar components [53]. It could also be that this MKK homologue regulates other promastigote functions during the attachment to host cells.

According to the central residue of the activation lip TXY motif [54], the TPY and TGY motifs are typical for stress-activated protein kinases (SAPKs) like p38. In *Leishmania* genome there are no typical SAPKs encoded with TPY and TGY motifs. However, two MAPK, LmxMPK4 and LmxMPK12 carrying a TQY motif could play a role of SAPKs in *L. mexicana* [52].

In conclusion, our results demonstrate that PKC-like among an ecto-PKC is important during *Leishmania* promastigote development through a stage-differential expression. These data suggest for the first time a direct link between PKC expression level and infectivity and provide evidence that this PKC-like plays a critical role in the attachment and in internalization steps of the invasion process. We hypothesize that, as a consequence of macrophage attachment, the PKC-like is over-expressed in order to phosphorylate other downstream signaling proteins such as SKK2-like and SAPK-like proteins. A similar pathway has been characterized in cancer models demonstrating a direct link between PKCα-regulated invasion in human carcinoma cells and p38 signaling [55].

It is important to note that the detection of several specific binding proteins with the microarray system let to predict other MAPK and PKCs with conserved domains compared to mammalian homologues. However, due to limitations associated to low expression levels in physiological conditions and the absence of specific antibodies, the real significance of these proteins remain to be confirmed genetic studies.

With the recent technological advances in the analysis of specific *Leishmania* proteins and their functions, new opportunities for drug development became possible. Hence, there seems to be welcome change in the attitude of pharmaceutical industry away from a strictly profit-oriented approach to drug development and towards a greater pursuit of drugs against nonprofitable diseases [56]. As with other parasitic diseases, there is no vaccine against leishmaniasis and chemotherapy involves toxicity and resistance [10]. The key role of *Leishmania*-PKC in the invasion process suggests that this enzyme could be a relevant therapeutic target for new anti-*Leishmania* drugs.
